# Acute Urinary Morbidity Following Stereotactic Body Radiation Therapy for Prostate Cancer with Prophylactic Alpha-Adrenergic Antagonist and Urethral Dose Reduction

**DOI:** 10.3389/fonc.2016.00122

**Published:** 2016-05-18

**Authors:** Michael C. Repka, Shan Guleria, Robyn A. Cyr, Thomas M. Yung, Harsha Koneru, Leonard N. Chen, Siyuan Lei, Brian T. Collins, Pranay Krishnan, Simeng Suy, Anatoly Dritschilo, John Lynch, Sean P. Collins

**Affiliations:** ^1^Department of Radiation Medicine, MedStar Georgetown University Hospital, Washington, DC, USA; ^2^Department of Pathology, MedStar Georgetown University Hospital, Washington, DC, USA; ^3^Department of Radiology, MedStar Georgetown University Hospital, Washington, DC, USA; ^4^Department of Urology, MedStar Georgetown University Hospital, Washington, DC, USA

**Keywords:** SBRT, CyberKnife, prostate cancer, quality of life, expanded prostate index composite, urethral dose reduction, AUA symptom score

## Abstract

**Background:**

Stereotactic body radiation therapy (SBRT) delivers high doses of radiation to the prostate while minimizing radiation to the adjacent critical organs. Large fraction sizes may increase urinary morbidity due to unavoidable treatment of the prostatic urethra. This study reports rates of acute urinary morbidity following SBRT for localized prostate cancer with prophylactic alpha-adrenergic antagonist utilization and urethral dose reduction (UDR).

**Methods:**

From April 2013 to September 2014, 102 patients with clinically localized prostate cancer were treated with robotic SBRT to a total dose of 35–36.25 Gy in five fractions. UDR was employed to limit the maximum point dose of the prostatic urethra to 40 Gy. Prophylactic alpha-adrenergic antagonists were initiated 5 days prior to SBRT and continued until resolution of urinary symptoms. Quality of life (QoL) was assessed before and after treatment using the American Urological Association Symptom Score (AUA) and the Expanded Prostate Cancer Index Composite-26 (EPIC-26). Clinical significance was assessed using a minimally important difference (MID) of one half SD change from baseline.

**Results:**

One hundred two patients underwent definitive prostate SBRT with UDR and were followed for 3 months. No patient experienced acute urinary retention requiring catheterization. A mean baseline AUA symptom score of 9.06 significantly increased to 11.83 1-week post-SBRT (*p* = 0.0024) and 11.84 1-month post-SBRT (*p* = 0.0023) but returned to baseline by 3 months. A mean baseline EPIC-26 irritative/obstructive score of 87.7 decreased to 74.1 1-week post-SBRT (*p* < 0.0001) and 77.8 1-month post-SBRT (*p* < 0.0001) but returned to baseline at 3 months. EPIC-26 irritative/obstructive score changes were clinically significant, exceeding the MID of 6.0. At baseline, 8.9% of men described their urinary function as a moderate to big problem, and that proportion increased to 37.6% 1 week following completion of SBRT before returning to baseline by 3 months.

**Conclusion:**

Stereotactic body radiation therapy for localized prostate cancer with utilization of prophylactic alpha-adrenergic antagonist and UDR was well tolerated as determined by acute urinary function and bother, and symptoms were comparable to those observed following conventionally fractionated external beam radiation therapy (EBRT). Longer follow-up is required to assess long-term toxicity and efficacy following SBRT with UDR.

## Introduction

Treatment of localized prostate cancer with definitive stereotactic body radiation therapy (SBRT) has become an accepted alternative to conventionally fractionated external beam radiation therapy (EBRT). However, acute urinary symptoms, such as frequency (1), weak stream (2), and dysuria (3), are common to both modalities during and after treatment due to the proximity of genitourinary structures (4). Patient comorbidities and large prostate volumes may increase the risk for symptoms (5–10), and neoadjuvant androgen deprivation may lessen their incidence and severity in patients treated with brachytherapy (11). Technical factors, such as the radiation dose to the bladder and urethra, as well as overall treatment time, may also impact the severity of such symptoms (12–15). Alpha-adrenergic antagonists are commonly used to manage urinary symptoms during and following radiation therapy (16), and clinical trials have suggested that prophylactic alpha-antagonists may decrease the peak severity and duration of urinary voiding symptoms following brachytherapy (17, 18).

The pattern of acute urinary toxicity following SBRT appears similar to that of conventionally fractionated EBRT and brachytherapy (19). Many patients suffer from acute urinary symptoms, whereas a minority of patients experience moderate to severe late symptoms (20). Following SBRT, acute urinary symptom scores peak 1 week after the completion of treatment (21) and return to baseline by 3 months. However, a minority of patients experience a clinically meaningful increase in their urinary symptoms >6 months after the completion of treatment (22). Late urinary symptom flare is associated with high bladder doses, inhomogeneous plans, and high prostatic urethral doses (23–25). This late urinary symptom flare resolves with conservative management (22).

Late urinary symptom flare may result from the high prostatic urethral doses seen in relatively inhomogeneous SBRT plans (20). Previous studies of urethral sparing with both EBRT and brachytherapy have demonstrated unacceptable levels of local recurrence (26). Urethral dose reduction (UDR) aims to reduce the risk of late urinary toxicity without significantly increasing the risk of local recurrence (27). Urethral catheter (UC) placement prior to simulation is a commonly used technique to identify the prostatic urethra. This approach is invasive, increases patient discomfort, and may increase the risk of iatrogenic urethral strictures. Furthermore, UC placement may distort the urethral anatomy, resulting in possible planning inaccuracies (28). Simple surrogates, such as a cylinder at the geometric center of the prostate, are especially inaccurate at the base (29). 3-T magnetic resonance imaging (MRI) provides an ideal non-invasive procedure to accurately identify the position of the urethra (30).

We have modified our institutional protocol to incorporate prophylactic alpha-adrenergic antagonists and limit the maximum urethral point dose to 110% of the prescription dose (40 Gy), as previously described for brachytherapy (31) with the aid of 3-T MRI. We aim to reduce the incidence and severity of urinary symptoms without a concomitant increased risk of local failure. In this study, we describe our current treatment approach and report our acute urinary toxicity outcomes for patients undergoing UDR with prophylactic alpha-adrenergic antagonist utilization.

## Materials and Methods

### Patient Selection

Patients eligible for study inclusion had prostate cancer treated with SBRT with prophylactic alpha-adrenergic antagonist use and UDR at MedStar Georgetown University Hospital. All patients provided informed consent prior to treatment. The Georgetown University Internal Review Board (IRB) approved this single institution prospective quality of life (QoL) study (IRB 12-1175).

### SBRT Treatment Planning and Delivery

Stereotactic body radiation therapy treatment planning and delivery were performed, as previously described (32) with modifications as follows. Gold fiducials were placed into the prostate under trans-rectal ultrasound guidance. For treatment planning, thin-cut computed tomography (CT) images were fused with high-resolution 3-T magnetic resonance (MR) images obtained without an endorectal coil. MR images were obtained using a 180-mm field of view, with an in-plane spatial resolution of 9 by 7 mm, and a through-plane spatial resolution of 3 mm. Accuracy of MR-CT fusion was verified by the treating physician (Sean P. Collins) prior to contouring or treatment planning. The prostatic urethra was identified on MR images using pre-contrast T2-weighted sequences and post-contrast T1-weighted sequences. The clinical target volume (CTV) included the prostate and proximal seminal vesicles. The planning target volume (PTV) included a 3-mm (inferior, superior, and posterior) or 5-mm (anterolateral) expansion around the CTV. A dose of 35–36.25 Gy was prescribed to the PTV in five fractions. The bladder, prostatic urethra, and membranous urethra were separately contoured and evaluated with dose–volume histogram (DVH) analysis to meet pre-specified planning objectives (Table 1) using Multiplan (Accuray Inc., Sunnyvale, CA, USA). The prostatic urethra was contoured on T2-weighted MRI sequences, and a planning risk volume (PRV) expansion was not employed. Treatment plans consist of hundreds of pencil beams using the IRIS variable aperture collimator (Accuray Inc., Sunnyvale, CA, USA). Plans were inhomogeneous by design to maximize dose to the prostate and to minimize dose to adjacent critical structures. The prescription isodose line was set at a minimum of 80% to limit the prostatic urethral dose such that the volume receiving 40 Gy was <0.03 cc. Radiation was delivered in treatments of approximately 40 min duration using the CyberKnife Radiosurgical System (Accuray Inc., Sunnyvale, CA, USA) every other day over the course of 11 days in total. Target position was confirmed multiple times during each treatment with a minimum of three properly placed fiducials (33). Prophylactic alpha-adrenergic antagonists were initiated 5 days prior to SBRT and continued until resolution of urinary symptoms. Bothersome urinary symptoms arising during or following treatment were managed with alpha-adrenergic antagonist dose increases from 0.4 to 0.8 mg daily if deemed necessary by the treating physician (Sean P. Collins). Refractory symptoms were treated with a short dexamethasone taper (2 mg daily for 7 days followed by 1 mg daily for 7 days).

**Table 1:** **Dose targets and constraints for treatment planning**.

36.25 Gy plan constraints	
PTV max dose	120% of 36.25 Gy (43.5 Gy)
PTV	V (36.25 Gy) ≥95%
CTV	V (36.25 Gy) ≥99%
Prostatic urethra	V (36.25 Gy) ≥95%
	V (40 Gy) ≤0.03 cc
Membranous urethra	V (37 Gy) <50%
Bladder	V (37 Gy) <5 cc
	V (100%) <10%
	V (50%) <40%
Rectum	V (36 Gy) <1 cc
	V (100%) <5%
	V (90%) <10%
	V (80%) <20%
	V (75%) <25%
	V (50%) <40%
Sigmoid colon	V (30 Gy) <1 cc
Penile bulb	V (29.5 Gy) <3 cc
Testicles	D (20%) <2 Gy

### Follow-up and Statistical Analysis

Overall health-related QoL was assessed at baseline using the EQ-5D survey with higher values indicating better QoL (34). Patients completed a paper questionnaire including the American Urological Association Symptom Index (AUA) (35) and the Expanded Prostate Cancer Index Composite-26 (EPIC-26) (36) on the day of the first SBRT fraction and during routine follow-up 1 and 3 months after the SBRT completion. One week following the completion of treatment, surveys were administered *via* phone interview by the study nurse practitioner (Thomas M. Yung). AUA scores range from 0 to 35 with higher values representing worsening urinary symptoms. To compare changes between time points, responses were assigned a score, and the significance of mean score changes was assessed by paired *t*-test. The EPIC-26 is a validated survey that measures urinary function and bother. Responses to the EPIC-26 questionnaire were grouped by physiologic domains and assigned numerical scores. The multi-item scale scores were transformed linearly to a 0–100 scale, as recommended in the scoring instructions for the EPIC-26. Lower numbers correspond to worse function and increased bother. The paired *t*-test and Wilcoxon signed-rank test were used to assess significance of the change in scores. For each physiologic domain question (Questions 4a–e) and the overall urinary bother question (Question 5), responses were grouped into three clinically relevant categories (no problem, very small to small problem, and moderate to big problem). Chi-square analysis was used to assess significance of proportional changes in each of the clinically relevant categories. The minimally important difference (MID), which is used to assess clinically significant change from baseline, was set as a half SD of the baseline mean (37).

## Results

**Figure 1:**
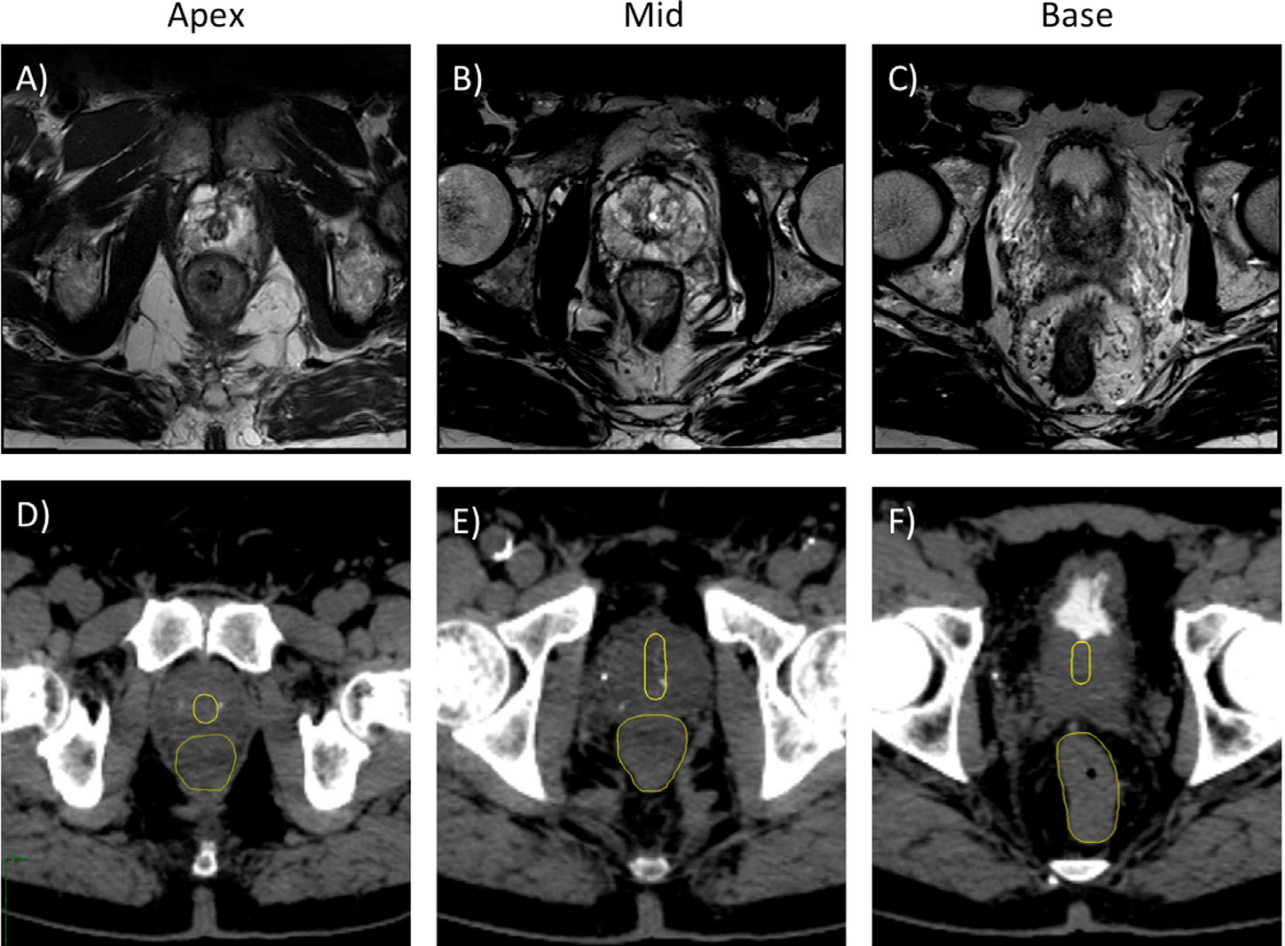
**Treatment planning axial T2-weighted MR (A–C) and axial CT (D–F) images demonstrating the prostatic urethra and rectum**. **(A,D)** Axial plane, 0.5 cm cranial to apex. **(B,E)** Axial plane, mid-prostate level. **(C,F)** Axial plane, 1 cm caudal to base.

**Figure 2:**
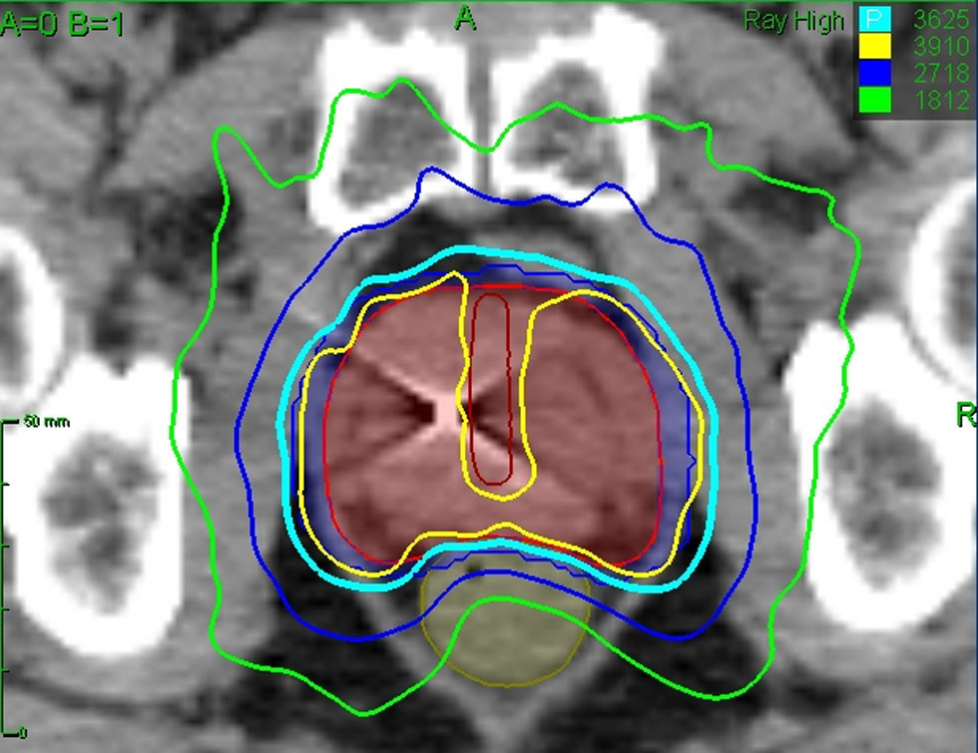
**Treatment planning axial CT images demonstrating the prostatic urethra (dark red line), prostate (red line), and rectum (brown line)**. Isodose lines shown are as follows: 108% of the prescription dose (yellow line), 100% of the prescription dose (cyan line), 75% of the prescription dose (dark blue line), 50% of the prescription dose (green line).

One hundred two patients with localized prostate cancer were treated with SBRT and followed for 3 months to evaluate acute toxicities. The median patient age was 69 years old (range: 48–84), and the median prostate volume was 36.0 cc (range: 16.6–125). Using the D’Amico risk classification, 17.6% of patients were low risk, 67.6% of patients were intermediate risk, and 14.7% patients were high risk. The majority of patients (63%) were treated with 36.25 Gy in 7.25 Gy fractions, while the remainder received 35 Gy in 7 Gy fractions (Table 2). The mean number of beams used for each fraction was 153.5 (range: 125–186) with a mean plan homogeneity index of 1.20 (range: 1.19–1.22). The mean prescription isodose line was 83.03% (range: 82–84%), and the mean planned fraction length was 39.05 min (range: 35–43 min). All patients met pre-specified planning objectives (Table 1). Representative 3-T MR and CT images show the prostatic urethra located centrally at the apex with anterior deviation as it approaches the bladder neck (Figures 1 and 2).

**Table 2:** **Baseline patient characteristics**.

Baseline characteristics		All (*n* = 102)
Age (years)	Median age (range)	69 (48–84)
Race	White	56.9%
	Black	30.4%
	Other	12.7%
Gleason score	6	29.4%
	7	59.8%
	8	9.8%
	9	1.0%
T stage	T1	66.7%
	T2	32.4%
	T3	1.0%
D’Amico risk group	Low	17.6%
	Intermediate	67.6%
	High	14.7%
Prostate volume	Median volume (cc) (range)	36.0 (16.6–125.0)
CCI	0	54.9%
	1	25.5%
	2+	19.6%
Pre-treatment PSA	Median PSA (ng/mL) (range)	7.15 (2.20–50.00)
Pre-treatment testosterone	Median testosterone (ng/dL) (range)	337 (3–990)
SBRT dose	35 Gy	36.6%
	36.25 Gy	63.4%
ADT	Yes	16.8%
	No	83.2%
AUA severity	Mild	46.1%
	Moderate	50.0%
	Severe	3.9%

**Figure 3:**
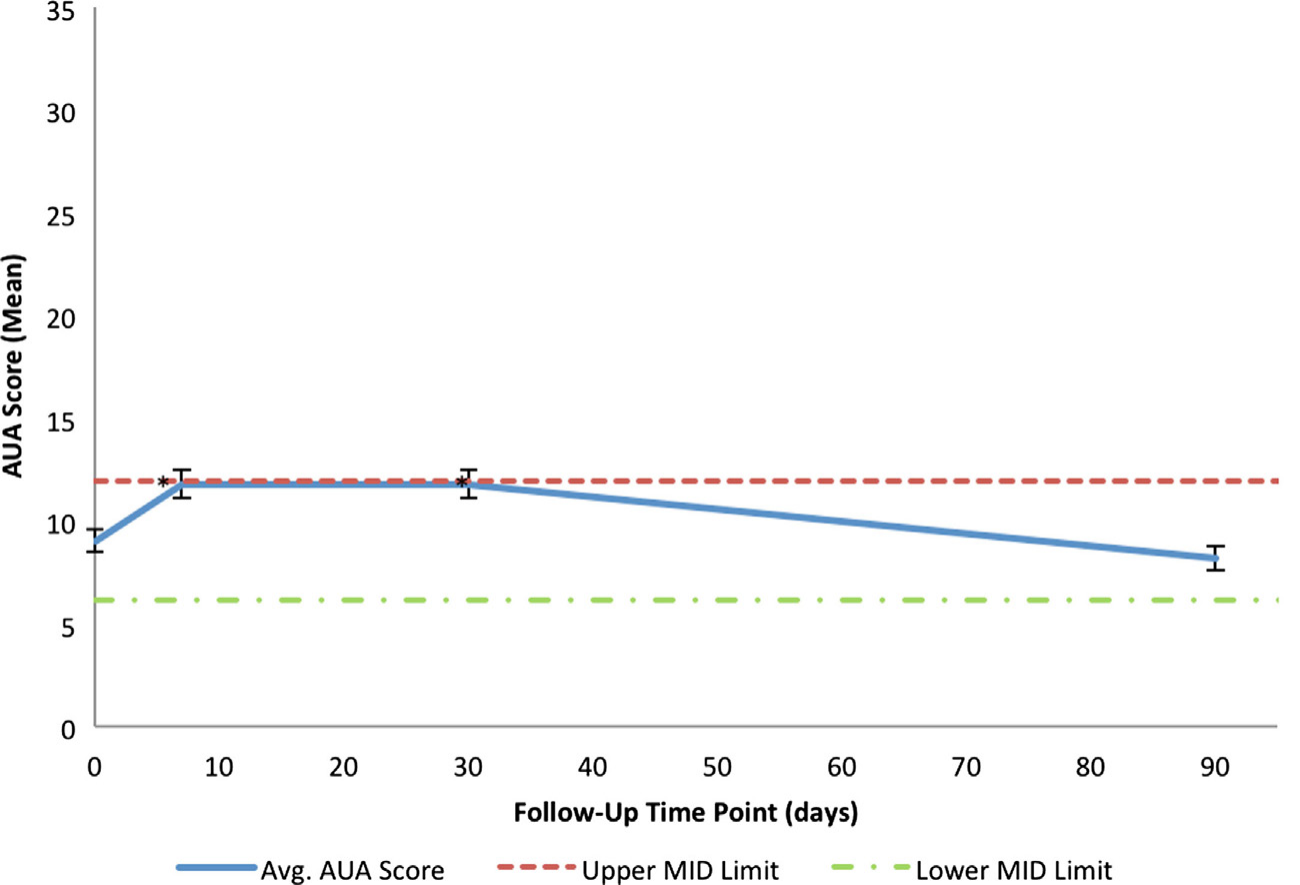
**Mean AUA scores in total at baseline and following SBRT for prostate cancer**. AUA scores range from 0 to 35 with higher values representing worse urinary symptoms. The thresholds for clinically significant changes in scores (half SD above and below the baseline) are marked with dashed lines. Error bars indicate 95% confidence intervals.

Consistent with a healthy elderly population, the mean baseline EQ VAS was 82.5 (Table 3). Baseline EPIC-26 urinary domain and AUA scores show that the majority of patients had mild to moderate lower urinary tract symptoms prior to receiving SBRT (Table 3). The mean AUA symptom score at 1 week and at 1 month post-treatment increased from 9.06 to 11.83 (*p* = 0.0024) and 11.84 (*p* = 0.0024), respectively (Table 4; Figure 3), although these statistically significant increases were of borderline clinical significance (MID = 2.87). The mean AUA score essentially returned to baseline at 3 months (Table 4; Figure 3).

**Table 3:** **Pre-treatment QoL scores**.

Pre-treatment quality of life (QoL) scores			
	Mean	SD	MID
**Baseline EQ-5D (*n* = 101)**
EQ-5D index	0.916	0.090	0.045
EQ VAS	82.5	14.29	7.14
**Baseline EPIC-26 urinary domains (*n* = 101)**
Incontinence	91.6	13.16	6.58
Irritative/obstructive	87.7	12.00	6.00
Bother	78.0	23.79	11.90
**Baseline AUA scores (*n* = 102)**
Total score	9.06	5.74	2.87

**Table 4:** **Changes in EPIC-2 urinary summary and overall urinary bother scores following SBRT for prostate cancer**.

Patient-reported quality of life from EPIC-26 urinary bother domains and AUA score									
	1 week post-treatment			1 month post-treatment			3 months post-treatment		
	Mean change	SD	***p***-Value	Mean change	SD	***p***-Value	Mean change	SD	***p***-Value
**EPIC-26 domain**	**(*n* = 101)**			**(*n* = 99)**			**(*n* = 88)**		
Incontinence	+2.2	13.80	0.0496*	−2.6	15.54	0.0429*	−0.65	13.55	0.2234
Irritative/obstructive	−13.6	17.40	<0.0001*	−9.9	15.96	<0.0001*	+0.18	11.27	0.2451
Urinary bother	−24.3	30.90	<0.0001*	−14.1	26.54	<0.0001*	−1.5	23.17	0.1193
**AUA score**	**(*n* = 102)**			**(*n* = 99)**			**(*n* = 88)**		
Total score	+2.73	7.1	0.0024*	+2.74	6.95	0.0024*	−0.9	5.4	0.2786

*Changes in scores that are statistically significant are marked with an asterisk*.

**Figure 4:**
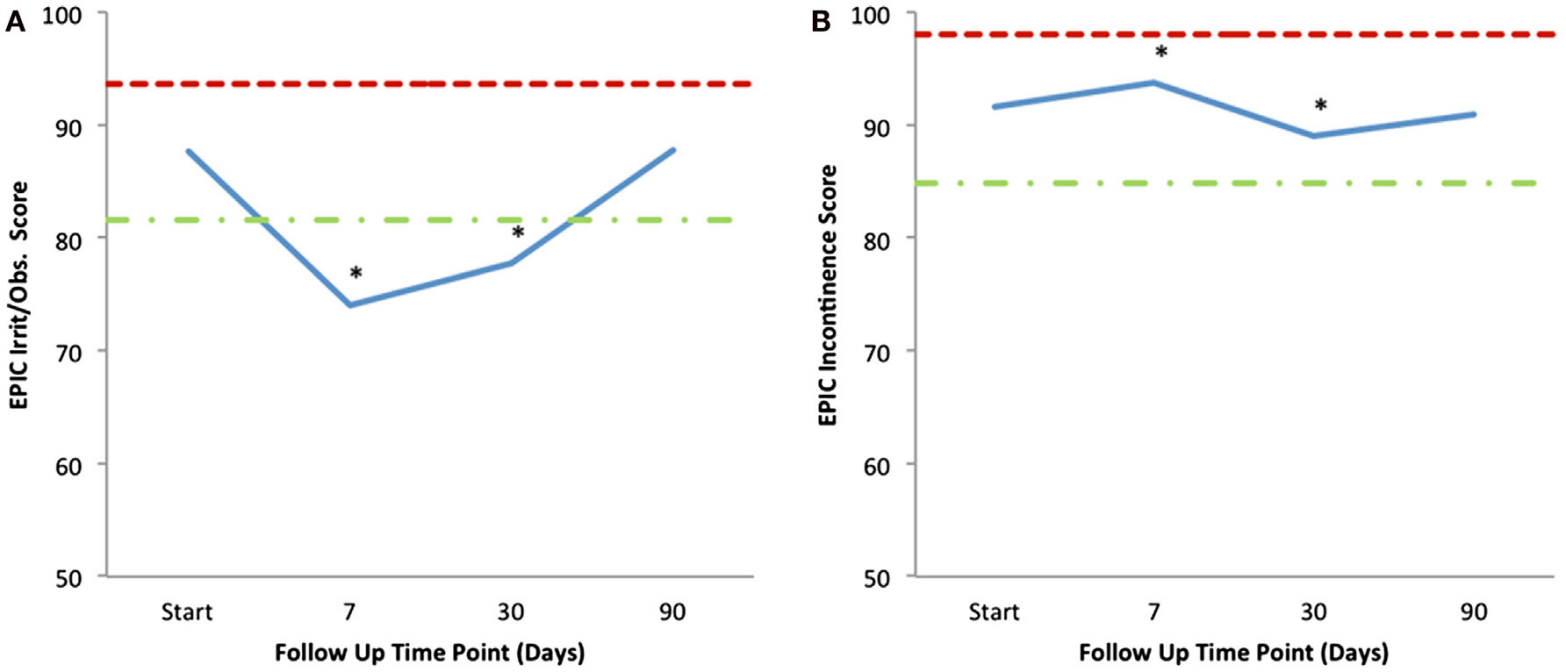
**Mean EPIC-26 urinary irritative/obstructive (A) and incontinence (B) scores following SBRT for prostate cancer**. EPIC-26 scores range from 0 to 100 with lower values representing worse symptoms. Thresholds for clinically significant changes in scores (half SD above and below the baseline) are marked with dashed lines.

**Figure 5:**
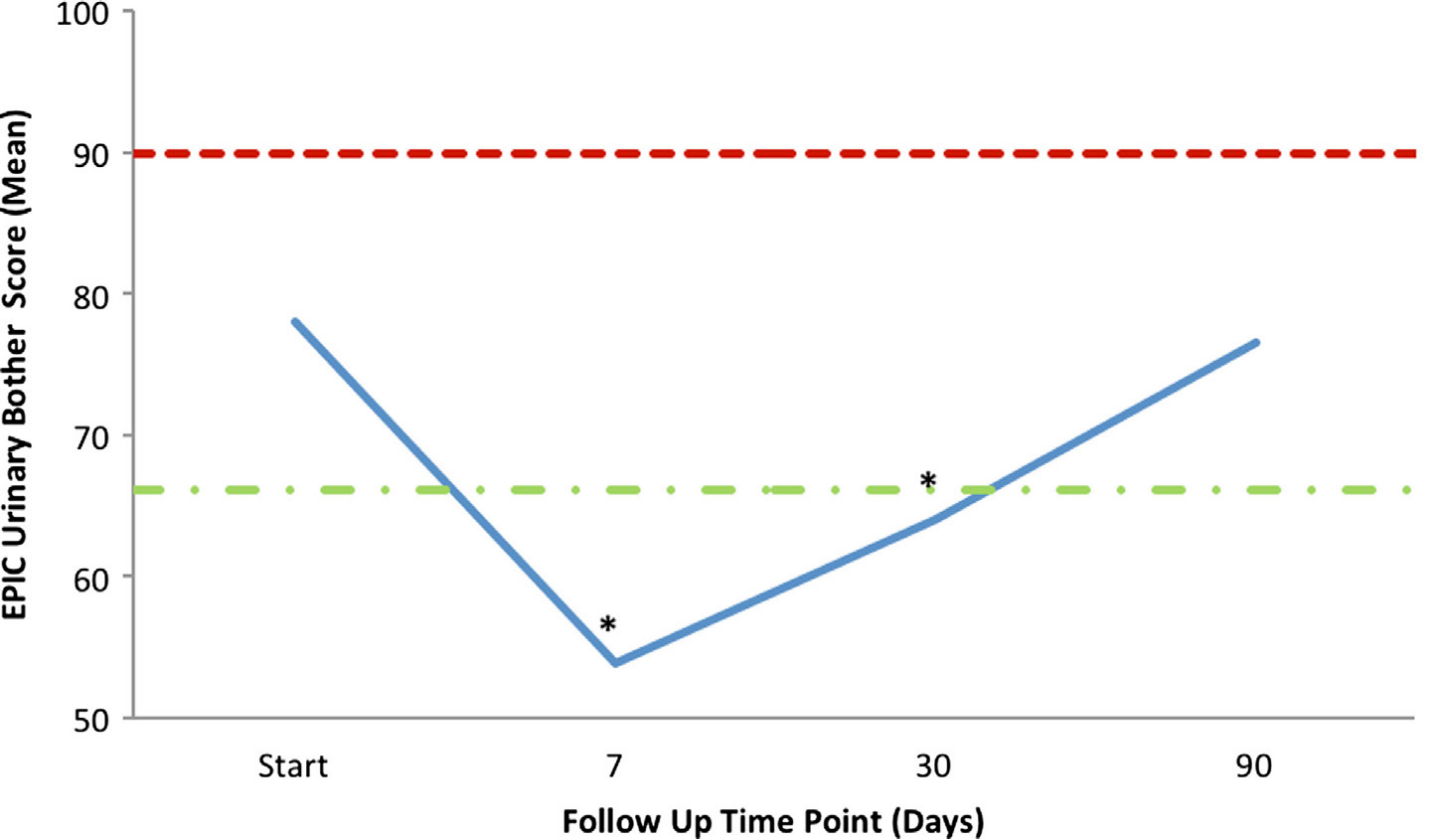
**Mean EPIC-26 overall urinary bother score (Question 5) following SBRT**. EPIC scores range from 0 to 100 with lower values representing worse symptoms. Thresholds for clinically significant changes in scores (half SD above and below the baseline) are marked with dashed lines.

Mean changes in EPIC-26 urinary domain-specific scores from baseline are shown in Table 4 and Figure 4. The EPIC-26 irritative/obstructive score decreased at 1 week and 1 month with a mean change of −13.6 (*p* < 0.0001) and −9.9 (*p* < 0.0001), respectively, post-SBRT. These declines were both statistically and clinically significant (MID = 6.0, Figure 4A). The EPIC irritative/obstructive score returned to baseline by 3 months (mean change = +0.18, *p* = 0.25) (Table 4; Figure 4A). There was no clinically significant change in the EPIC incontinence score during the first 3 months following SBRT (Table 4; Figure 4B). The EPIC urinary bother score declined transiently at 1 week and 1 month, with mean changes of −24.3 (*p* < 0.0001) and −14.1 (*p* < 0.0001), respectively, following treatment (Table 4). These declines were both statistically and clinically significant (MID = 11.9). By 3 months post-SBRT, clinically significant changes had resolved, as the EPIC urinary bother score returned to baseline levels (mean change = −1.5, *p* = 0.12) (Table 4; Figure 5).

**Figure 6:**
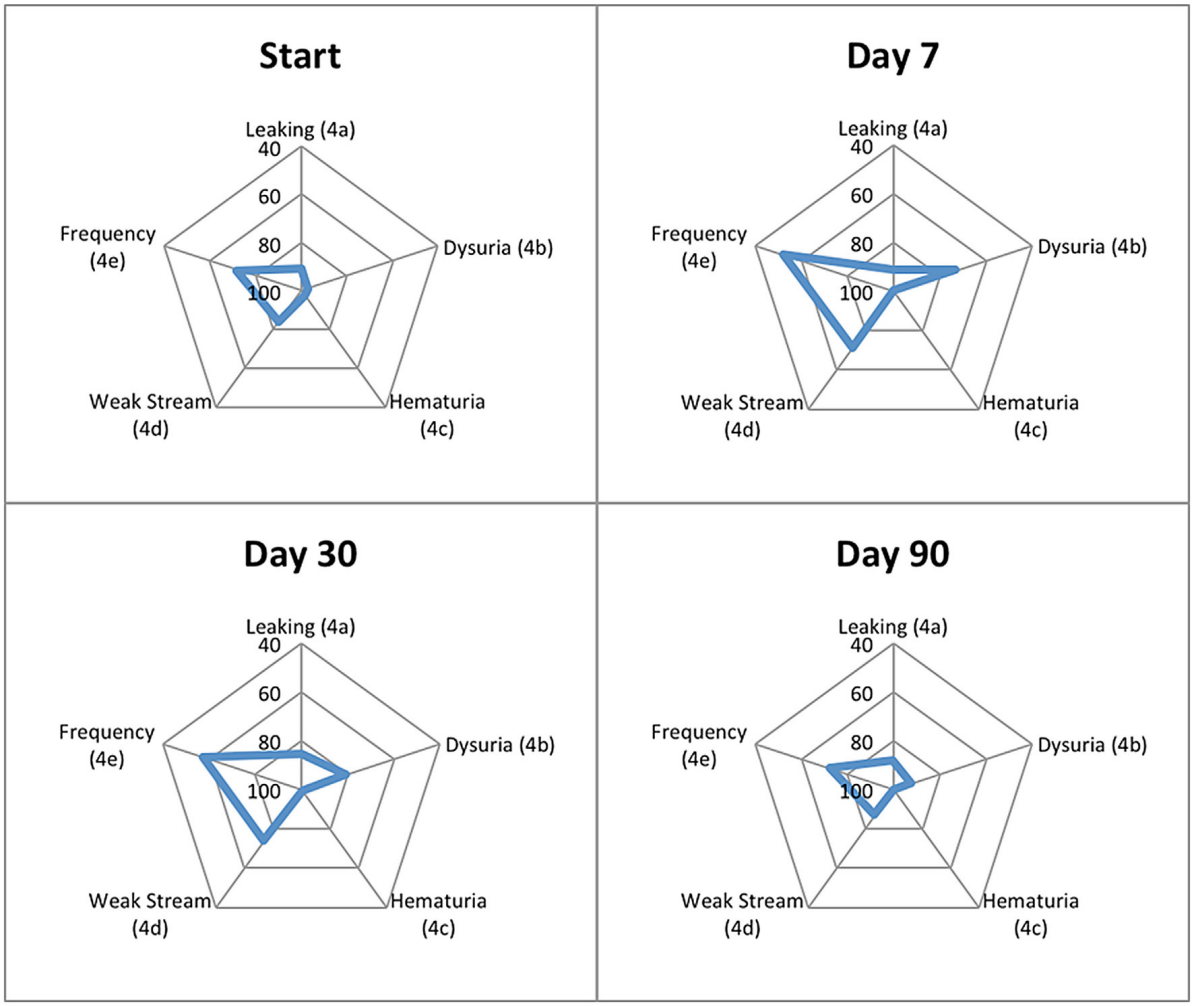
**Radar plots showing the distribution of individual symptom bother following SBRT for prostate cancer**. EPIC-26 scores range from 0 to 100 with lower values representing worse symptoms. Points further from center indicate higher levels of bother for a given symptom. EPIC-26 question number is shown in parentheses.

Patient-reported responses for urinary incontinence and hematuria did not show clinically significant changes from baseline, while urinary retention and urinary frequency increased significantly 1-week and 1-month post-SBRT but returned to baseline by 3 months (Table 5). Dysuria increased significantly and improved, but did not return to baseline, by 3 months following completion of SBRT (Table 5). Regarding overall urinary bother (Question 5 of the EPIC-26), 50.5% of our cohort reported some level of bother due to urinary symptoms, and 8.9% of patients reported that urinary symptoms were a moderate to big problem. One week and 1 month following SBRT, 37.6% (*p* < 0.0001) and 18.2% (*p* < 0.0002) of our cohort reported that overall urinary bother was a moderate to big problem (Table 6). Patient-reported urinary bother returned to baseline by 3 months, with 8.0% (*p* = 0.66) of the cohort reporting a moderate to big problem. Radar plots summarize the time course of individual symptom bother before and after SBRT in Figure 6. Urinary frequency and weak stream bother peak at 1 week and gradually return to baseline by 3 months following completion of SBRT, while dysuria peaks at 1 week and improves but does not return to baseline by 3 months. Table 7 summarizes urinary morbidity data at 1 and 3 months for relevant brachytherapy and SBRT series.

**Table 5:** **Urinary symptoms following SBRT**.

	Baseline	1 week	1 month	3 months
	
Number (*n*)	101	101	99	88
**Urinary incontinence**				
No problem (%)	71.3	81.2	60.6	62.5
Very small-small problem (%)	27.7	13.9	35.4	36.4
Moderate-big problem (%)	1.0	5.0	4.0	1.1
*p*-Value		0.6420	0.0066*	0.0637
**Dysuria**				
No problem (%)	92.1	46.5	51.5	75.0
Very small-small problem (%)	5.9	36.6	41.4	25.0
Moderate-big problem (%)	2.0	16.8	6.1	0.0
*p*-Value		<0.0001*	<0.0001*	0.0039*
**Hematuria**				
No problem (%)	94.0	100.0	96.0	98.9
Very small-small problem (%)	5.0	0.0	4.0	1.1
Moderate-big problem (%)	1.0	0.0	0.0	0.0
*p*-Value		0.0313*	0.3750	0.1250
**Urinary retention**				
No problem (%)	56.4	48.5	40.4	55.7
Very small-small problem (%)	40.6	33.7	45.5	43.2
Moderate-big problem (%)	3.0	17.8	14.1	1.1
*p*-Value		0.0002*	0.0002*	0.7713
**Urinary frequency**				
No problem (%)	33.7	24.8	19.2	35.2
Very small-small problem (%)	53.5	33.7	53.5	51.1
Moderate-big problem (%)	12.9	41.6	27.3	13.6
*p*-Value		<0.0001*	<0.0001*	0.7246

*Patient-reported responses to EPIC-26 questions 4A (dripping), 4B (dysuria), 4C (Hematuria), 4D (weak stream or incomplete emptying), and 4E (frequency)*.

*Changes in scores that are statistically significant are marked with an asterisk*.

**Table 6:** **Overall urinary bother following SBRT**.

	Baseline	Day 7	Month 1	Month 3
	
Number (***n***)	101	101	99	88
**Urinary bother**
No problem (%)	40.6	19.8	21.2	35.2
Very small–small problem (%)	50.5	42.6	60.6	56.8
Moderate-big problem (%)	8.9	37.6	18.2	8.0
*p*-Value		<0.0001*	0.0002*	0.6567

*Patient-reported responses to EPIC-26 Question 5*.

*Changes in scores that are statistically significant are marked with an asterisk*.

**Table 7:** **Comparison of published AUA score changes following brachytherapy and SBRT**.

Reference	*N*	Modality	Total dose (Gy)	No. of fractions	Urethral dose	Baseline AUA score	1-week AUA score	1-month AUA score	3-month AUA score	Long-term AUA score
Henderson et al. (38)	255	LDR ± EBRT	145 (110 + 45)	n/a	Mean D10 = 232 Gy	6	n/r	19 (6 weeks)	15	5 (2 years)
Van Gellekom et al. (39)	127	LDR	D90 = 133.0 (mean)	n/a	Mean D5 = 270 Gy	7.3	n/r	19.8	15.2 (6 months)	10.4 (2 years)
Williams et al. (40)	173	LDR	D90 = 136.5 (median)	n/a	Mean *D*_max_ = 219 Gy	5.5	n/r	17.1	14.4	8.0 (1 year)
Crook et al. (10)	150	LDR	145	n/a	Mean *D*_max_ = 209 Gy	6	n/r	13	13	3 (2 years)
Meier et al. (41)	295	SBRT	40	5	n/r	7.6	14.2	11.6	7.5	6.4 (4 years)
Tree et al. (42)	51	SBRT	36.25	5	n/r	6	11 (1–3 weeks)	8 (4–6 weeks)	5 (7–12 weeks)	n/r
Rana et al. (43)	102	SBRT	35–40	5	n/r	10.5	n/r	13.4	10.6	8.6 (3 years)
Present study	102	SBRT	35–36.25	5	*D*_max_ < 40 Gy	9.1	11.8	11.8	8.2	n/r

*n/r, not reported; n/a, not applicable*.

## Discussion

Most patients develop acute voiding symptoms which peak 1–2 weeks following SBRT for localized prostate cancer (20). Although evidence for early urinary morbidity is growing (44), this study is among the first to comprehensively assess urinary morbidity at 1 week following SBRT. We have prospectively employed several approaches to minimize urinary symptoms following SBRT. The overall treatment time has been extended from 5 to 11 days (15), prophylactic alpha-adrenergic antagonist use was initiated prior to treatment, and the maximum point dose to the prostatic urethra was limited to <40 Gy. Our prior treatment approach allowed for central prostatic point doses up to 45 Gy (20). Given reports of high rates of treatment failure with urethral-sparing approaches (26), we designed our current UDR approach to maintain excellent disease control while reducing late urinary toxicity.

In this study, we report a small statistically significant increase in the AUA score 1 week following SBRT. However, the increase of approximately three points was of borderline clinical significance and compares favorably with prior trials (21). Consistent with previous reports from our institution (20) and others (45), the mean AUA score remained elevated 1-month post-treatment and returned to baseline by 3 months. It is significant that the peak rise and duration of post-SBRT AUA score elevations was less than reported for patient receiving prophylactic alpha agonists for low-dose rate (LDR) brachytherapy (18, 46). Furthermore, these results indicate that QoL outcomes at 1 week are similar to those at 1 month following completion of SBRT, suggesting that early follow-up is redundant. Despite an apparent lack of utility for research purposes, we have maintained the institutional policy to call patients 1 week following SBRT to optimize medical management and provide reassurance if needed.

We have reported an SBRT-induced increase in acute urinary irritative and obstructive symptoms with little impact on urinary continence. The EPIC irritative/obstructive score decreased at 1 week and 1 month following treatment, with a return to baseline by 3 months. There was no clinically significant change in the EPIC incontinence domain during the first 3 months following SBRT. Although dysuria increased significantly from baseline and did not resolve at 3 months following SBRT, anecdotal clinical evidence suggests that dysuria tends to take more time to resolve than other urinary symptoms, which may account for this observation. However, overall urinary bother may be more important to the patient than specific urinary symptoms. We found that moderate to severe overall urinary bother increased following SBRT from a baseline of 8.9–37.6% 1 week following treatment. As seen with conventionally fractionated EBRT, this increase in urinary bother decreased at 1 month and returned to baseline by 3 months following completion of treatment (19).

Urethral dose reduction does not eliminate acute urinary morbidity following SBRT, and dose effects on other genitourinary structures may serve as better predictors of acute urinary toxicity. In a study of patients undergoing LDR brachytherapy, bladder neck D2 cc was a better predictor of acute urinary toxicity than typically employed dose–volume constraints for the prostatic urethra (14). However, the goal of UDR is to improve long-term patient QoL, specifically by mitigating urinary symptom flare occurring at 12 months post-SBRT (20, 22). With longer follow-up of this patient cohort, we will examine the effects of UDR on the incidence and severity of late urinary symptom flare. Future studies should further illuminate the nature of urinary morbidity secondary to prostatic urethral dose. For example, RTOG 0938, a multi-institutional randomized trial of SBRT versus moderate hypofractionation, required limitation of the urethra to 107% of prescription dose. Our report, in conjunction with other emerging data (44), portend well for continued improvement in the therapeutic ratio for prostate SBRT.

Recently reported results from the PATRIOT trial, which randomized patients to an every-other-day (11 days in total) or once-weekly (29 days in total) fractionation scheme, suggest that extension of total treatment time to 1 month may decrease acute urinary morbidity (47). In our experience, extending overall treatment times beyond 2 weeks is unnecessary and may be burdensome to some patients. Importantly, the PATRIOT trial did not show an increase in irreversible late morbidity in the 11-day group. Furthermore, our 11-day treatment regimen was well tolerated with no patient requiring catheterization; patients’ symptoms were short lived and resolved with conservative medical management. Our study shows that an 11-day SBRT treatment course with prophylactic alpha-adrenergic antagonist use and UDR is well tolerated with regard to acute urinary toxicity. Further follow-up of this cohort will be necessary to evaluate late urinary toxicity and ensure equivalent treatment efficacy to SBRT without UDR for localized prostate cancer.

## Conclusion

Stereotactic body radiation therapy for clinically localized prostate cancer with UDR and prophylactic alpha-adrenergic antagonist use was well tolerated with acute urinary function and bother comparable to conventionally fractionated EBRT. In the first 3 months following treatment, the impact of SBRT on urination was minimal. Longer follow-up will be required to assess long-term morbidity and efficacy following SBRT with UDR.

## Author Contributions

MR, SG, and RC are the lead authors who participated in data analysis, manuscript drafting, table/figure creation, and manuscript revision. TY performed quality of life data collection. HK and LC aided in the quality of life data analysis and manuscript revision. BC participated in the design and coordination of the study. PK provided radiology guidance and aided in figure creation and manuscript revision. SS aided in quality of life analysis and manuscript revision. AD is a senior author who aided in drafting the manuscript. JL is a senior author who aided in drafting the manuscript. SC is the principal investigator who conceived the study design and participated in data collection, data analysis, table/figure creation, manuscript drafting, and manuscript revision. All authors read and approved the final manuscript.

## Conflict of Interest Statement

SP Collins and BT Collins serve as clinical consultants to Accuray Inc. The Department of Radiation Medicine at Georgetown University Hospital receives a grant from Accuray to support a research coordinator. The other authors declare that they have no competing interests.

## Abbreviations

3-T, 3-Tesla; ADT, androgen deprivation therapy; AUA, American Urological Association; CTV, clinical target volume; DIL, dominant intraprostatic lesion; DVH, dose–volume histogram; EBRT, external beam radiation therapy; EPIC-26, expanded prostate index composite; IMRT, intensity-modulated radiation therapy; LDR, low-dose rate; PET, positron emission tomography; PTV, planning target volume; QoL, quality of life; SBRT, stereotactic body radiation therapy.
